# Expert consensus on the management of nocturnal enuresis in children

**DOI:** 10.1007/s12519-026-01051-4

**Published:** 2026-07-10

**Authors:** Qian Shen, Hong Xu, The Cooperative Group Member, Qian Shen, Hong Xu, Bing An, Ying Bao, Chao-Ying Chen, Xi-Qiang Dang, Mei-Li Fan, Rui Fu, Li Fu, Wei-Hua Gan, Xia Gao, Xiao-Jie Gao, Fang-Qi Hu, Peng Hu, Xiao-Yun Jiang, Yu-Lin Kang, Guo-Min Li, Xiao-Zhong Li, Li-Jun Liang, Yi Lin, Ya-Lan Liu, Cui-Hua Liu, Xiao-Mei Liu, Yu-Ling Liu, Xue-Yun Lv, Jun Ma, Jian-Hua Mao, Li Miao, Qian-Fan Miao, Xiao-Jing Nie, Zan-Hua Rong, Xiao-Shan Shao, Qian Shen, Tong Shen, Qing Sun, Shu-Zhen Sun, Yu-Hong Tao, De-Xuan Wang, Fei-Yan Wang, Mo Wang, Xiao-Wen Wang, Wen-Hong Wang, Fu-Jie Wen, Yu-Bin Wu, Fu-Jie Wen, Jian-Wen Wu, Zheng-Kun Xia, Hong Xu, Yong Yao, Zhu-Wen Yi, Zhao-Qing Yin, Li Yu, Lei Yu, Dong-Feng Zhang, Jian-Jiang Zhang, Fei Zhao, Li-Ping Zhao, Li-Jun Zhao, Bo Zhao, Ping Zhou, Wei-Ran Zhou, Jian-Hua Zhou, Da-Qian Zhu, Ying Zhu

**Affiliations:** Department of Nephrology, Children’s Hospital of Fudan University, National Children’s Medical Center, Shanghai, 201102 China

**Keywords:** Adolescents, Children, Monosymptomatic, Nocturnal enuresis

## Abstract

**Background:**

Nocturnal enuresis (NE) is a common condition in childhood that can adversely affect children’s physical and psychological health. Since the publication of the “Expert Consensus on the Management of Monosymptomatic Nocturnal Enuresis in Chinese Children” in 2014, substantial clinical experience has been accumulated and international guidelines have been updated, necessitating a revision to standardize and improve clinical management.

**Methods:**

This consensus was developed by the Chinese Cooperative Group for the Management of Pediatric Nocturnal Enuresis, the Pediatric Nephrology Committee of the Chinese Medical Doctor Association using the Delphi method. A core writing group of nine experts drafted statements based on a synthesis of current evidence. These were subjected to iterative voting by 42 multidisciplinary panelists to achieve consensus, defined as ≥ 90% agreement.

**Results:**

The consensus provides 18 key recommendations. It updates the diagnostic criteria for children aged ≥ 5 years with at least one involuntary void per month for three months. It emphasizes classification into monosymptomatic (MNE) or nonmonosymptomatic (NMNE) NE based on daytime lower urinary tract symptoms. Recommendations cover comprehensive evaluation (history, physical exam, voiding diary), basic management (education, lifestyle), and first-line treatment (desmopressin for nocturnal polyuria, alarm therapy for reduced bladder capacity). Strategies for treatment-resistant cases, management of NMNE (prioritizing daytime symptoms), and the role of primary care and traditional Chinese medicine are outlined.

**Conclusions:**

This expert consensus provides an updated, evidence-based, and practical framework for the diagnosis and management of childhood NE. It aims to harmonize clinical practice, promote individualized care, and improve patient outcomes through standardized recommendations.

**Graphical abstract:**

**Figure:**
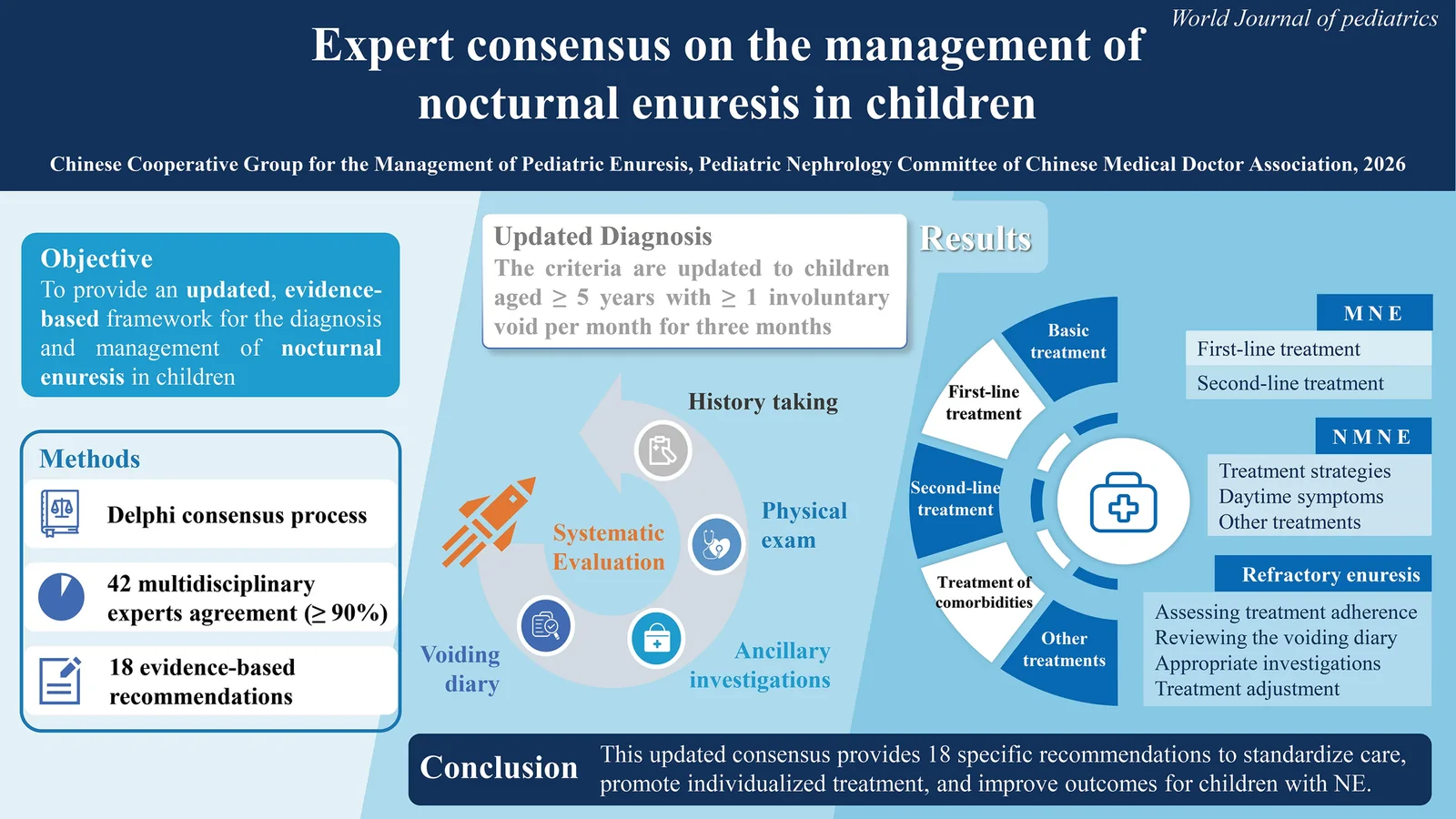

**Supplementary Information:**

The online version contains supplementary material available at https://doi.org/10.1007/s12519-026-01051-4.

## Introduction

Nocturnal enuresis (NE) is a prevalent pediatric disorder characterized by intermittent incontinence during sleep. It imposes a significant burden, adversely affects children’s self-esteem, emotional well-being, social functioning, overall quality of life and family life. The etiological understanding of NE has evolved substantially, recognizing a multifactorial basis often involving nocturnal polyuria, reduced bladder capacity, and arousal dysfunction. Despite the availability of effective treatments, significant challenges persist in the routine clinical management of NE globally, including underdiagnosis, heterogeneous practice patterns, and suboptimal treatment adherence. Within the specific context of China, additional factors—such as regional disparities in healthcare access, varied cultural perceptions of the condition, and the need for integrated care across different levels of the healthcare system—create a distinct clinical landscape. In 2014, the Chinese Cooperative Group for the Management of Pediatric Enuresis, Pediatric Nephrology committee of Chinese Medical Doctor Association published the *“*Expert Consensus on the Management of Monosymptomatic Nocturnal Enuresis in Chinese Children*”* [1] (hereafter referred to as the “2014 version”), which served as a reference and guide for clinicians. However, the field has progressed considerably since then: new evidence has emerged, international standards have been updated [2–10], and a wealth of domestic clinical experience has accumulated. This has created a gap between contemporary best practices and frontline clinical care in China, necessitating an updated, pragmatic, and standardized framework tailored to the local healthcare environment. Therefore, the Chinese Cooperative Group for the Management of Pediatric Enuresis, Pediatric Nephrology committee of Chinese Medical Doctor Association revised and updated the 2014 version to promote the standardization of clinical practice for pediatric enuresis. The novelty and significance of this consensus are threefold: (1) employing a formal Delphi process with a national multidisciplinary panel to ensure practical applicability; (2) explicitly integrating international evidence with the realities of the Chinese healthcare setting; and (3) delivering updated, actionable, and comprehensive recommendations for the diagnosis, classification, and individualized management of NE to standardize and improve care across China.

## Methods

### Expert panel

The consensus was developed by the Chinese Cooperative Group for the Management of Pediatric Enuresis and the Pediatric Nephrology Committee of the Chinese Medical Doctor Association. A core writing group was first formed, consisting of nine experts and one secretary. The members were Prof. Hong Xu and Prof. Qian Shen, Prof. Xiaoyun Jiang, Prof. Xiaomei Liu, Prof. Cuihua Liu, Prof. Jianhua Mao, Prof. Xiaoshan Shao, Prof. Tong Shen, and Prof. Zhengkun Xia. This group, led by Prof. Hong Xu was responsible for systematic literature retrieval, screening, evidence appraisal, and identification of key clinical issues. The core writing group collectively handled the conceptualization, methodology design, and the original drafting and subsequent reviewing or editing of the manuscript. Dr. Qianfan Miao served as the secretary, responsible for consolidating the initial drafts and participating in the review and editing process. The broader multidisciplinary consensus panel comprised the 42 core members of the Chinese Cooperative Group for the Management of Pediatric Enuresis, representing pediatric nephrology, urology, child healthcare, and traditional Chinese medicine from across China. This panel was responsible for the validation, formal analysis, and consensus-building through structured voting and iterative rounds. All members of the Collaborative Group contributed to critical review and final consensus approval.

### Consensus development process

This consensus was developed using internationally accepted Delphi methodology [11]. The specific steps were: (1) a core writing group consisting of nine enuresis experts and one secretary was established. Members were divided into three working groups, diagnosis group, monosymptomatic treatment group, and nonmonosymptomatic treatment group, to perform literature retrieval, screening, and appraisal and to identify key clinical issues using the patient, intervention, comparison, outcome (PICO) framework, requiring attention or revision in the consensus; (2) each working group member, based on evidence from guidelines, consensus statements, systematic reviews, meta-analyses, and clinical studies, discussed in meetings and communicated extensively with international experts. They then drafted statements for each PICO question along with recommended consensus points and evidence interpretation for their respective sections. These were compiled by the secretary into a first draft of the consensus; (3) a total of 42 experts in the enuresis cooperative group voted on each recommendation via an electronic questionnaire. The core writing group was composed of nine pediatric nephrologists, while the 42 experts included multidisciplinary experts from pediatric nephrology, pediatric urology, child healthcare, and pediatrics in traditional Chinese medicine. The voting options were as follows: (1) fully agree (essential, minimum requirement); (2) partially agree or recommended (desirable but not absolutely essential), and (3) do not agree (should be removed; unreasonable, unnecessary, not suitable for the national context, not feasible or not evaluable, and thus not to be included in the consensus). For options (2) and (3), experts were required to provide a rationale and suggestions for revision. A recommendation was considered to have reached expert consensus if more than 75% of the experts selected “fully agree”; (4) voting results were compiled, and the draft was revised and supplemented according to the experts’ opinions before proceeding to the next round of voting. This process was repeated until consensus was achieved. Finally, the secretary collated the experts’ feedback to form the final version of the consensus. A total of three rounds of voting were performed, resulting in 18 recommendations, all achieving consensus (each recommendation had over 90% “fully agree”).

## Recommendations and discussion

### Diagnosis of nocturnal enuresis

#### Definition and classification

The definition of NE is intermittent urinary incontinence during nighttime sleep. The diagnostic criteria have varied among different countries and organizations with the passage of time and increasing awareness of NE. The 2014 version defined pediatric enuresis as involuntary nighttime urination occurring in children aged ≥ 5 years, with a frequency of at least two episodes per week for more than three months, according to the Diagnostic and Statistical Manual of Mental Disorders (DSM-IV), and emphasized that the diagnostic criteria could be applied more flexibly in older children [1]. The International Children’s Continence Society (ICCS) subsequently updated its definition to children aged at least five years, with at least one episode of involuntary urination during sleep per month, for a duration of three months in 2014 [4]. Later on, ICCS guideline updates in 2016 and 2020 maintained this definition [5, 6]. The criterion of age ≥ 5 years reflects the developmental level of urinary control. A frequency of at least one episode per month for a minimum of three months facilitates exclusion of occasional enuresis due to factors such as fatigue or excessive pre-bedtime fluid intake. In recent years, with socioeconomic development and increased public health awareness, “bedwetting” is highlighted. Even infrequent bedwetting in school-age children may cause significant concern and a desire for treatment from parents and children. Furthermore, untreated enuresis can lead to psychosocial problems, affecting the child’s physical and mental health, social life, academic performance, and sleep, while also imposing a burden and psychological stress on the child and family [12, 13]. A large epidemiological survey in China revealed the following prevalence rates among 5-year-old children: 15.2% when defined as at least one bedwetting episode in three months, 7.9% for at least one episode per month, and 4.5% for at least two episodes per week [14]. Updating the diagnostic criteria for enuresis in line with international authoritative guidelines facilitates earlier diagnosis and, depending on the condition and the child’s and family’s wishes, allows for active treatment to reduce the impact of the disease.

NE can be classified in different ways, but clinically, the primary distinction is based on the presence or absence of daytime lower urinary tract symptoms (LUTS). Thus, enuresis is categorized as either monosymptomatic enuresis (MNE) or non-monosymptomatic enuresis (NMNE). The key diagnostic points for MNE are: (1) age older than five years; (2) involuntary urination during sleep at least once per month for more than three months; and (3) no accompanying daytime LUTS. The diagnostic criteria for NMNE are the presence of one or more daytime LUTS (including daytime incontinence, increased voiding frequency, urgency, decreased voiding frequency, an intermittent stream, hesitancy, or voiding difficulty) in addition to meeting (1) and (2) above. Enuresis can also be described based on the presence or absence of a previous dry period of six months or longer, dividing it into primary nocturnal enuresis (PNE) and secondary nocturnal enuresis (SNE). Definitions of terms related to enuresis are shown in Table 1 [1, 5, 6].

**Table 1:** Terminology related to nocturnal enuresis

Terminology	Definition
Nocturnal enuresis	Involuntary nighttime voiding occurring at least once per month for at least three months in children aged 5 y or older
Monosymptomatic nocturnal enuresis	Nocturnal enuresis in the absence of any daytime lower urinary tract symptoms
Non-monosymptomatic nocturnal enuresis	Nocturnal enuresis accompanied by daytime lower urinary tract symptoms
Primary nocturnal enuresis	Enuresis persisting since early childhood, without a continuous dry period of 6 mon or longer, and in the absence of organic disease
Secondary nocturnal enuresis	Recurrence of enuresis after a sustained dry period of 6 mon or longer
Expected bladder capacity	For children < 12 y: [(age in y + 1) × 30] mL; for age 12–18 y: 390 mL
Maximum bladder volume	The largest single voided volume recorded within a 24 h period (excluding the first morning void), as documented in a bladder diary for at least 2 day
Total voided volume	The combined volume of urine produced during the night, including involuntary or voluntary voids during sleep (measured by diaper weight gain or collected volume) and the first morning void
Nocturnal polyuria	Nocturnal urine output exceeding 130% of the expected bladder capacity for the age of child
Overactive bladder	A symptom syndrome characterized by urgency, with or without urge incontinence
Daytime lower urinary tract symptoms	Daytime symptoms including incontinence, increased voiding frequency, urgency, decreased voiding frequency, an intermittent stream, hesitancy, or voiding difficulty
Urinary incontinence (leakage)	The involuntary loss of urine during the daytime
Increased voiding frequency	Daytime voiding at least eight times per day
Urgency	A sudden, compelling desire to void that is difficult to defer
Decreased voiding frequency	Daytime voiding less than four times per day
Voiding difficulty	Symptoms including hesitancy (delay in initiating voiding), weak stream, straining, or the need for abdominal pressure to aid voiding

This article has been previously published in *Journal of Clinical Pediatrics*, 2025, Volume 43, Issue 7, 2025, pages 483–499 (https://doi.org/10.12372/jcp.2025.25e0564); under the title “Chinese expert consensus on the management of nocturnal enuresis in children (2025)” in Chinese. *The Journal of Clinical Pediatrics* has graciously agreed to transfer the copyright of the English version of the “Chinese expert consensus on the management of nocturnal enuresis in children (2025)” to the *World Journal of Pediatrics*. *The Journal of Clinical Pediatrics* is an open access journal

**Recommendation 1:** The definition of pediatric NE is updated to children aged five years or above with at least one involuntary urination during sleep per month on average, lasting for three months or more (fully agreed: 97.5%).

**Recommendation 2:** After confirming a diagnosis of NE, classify it as MNE or NMNE based on the presence or absence of daytime LUTS (fully agreed: 97.5%).

#### Evaluation of nocturnal enuresis

**Fig. 1:**
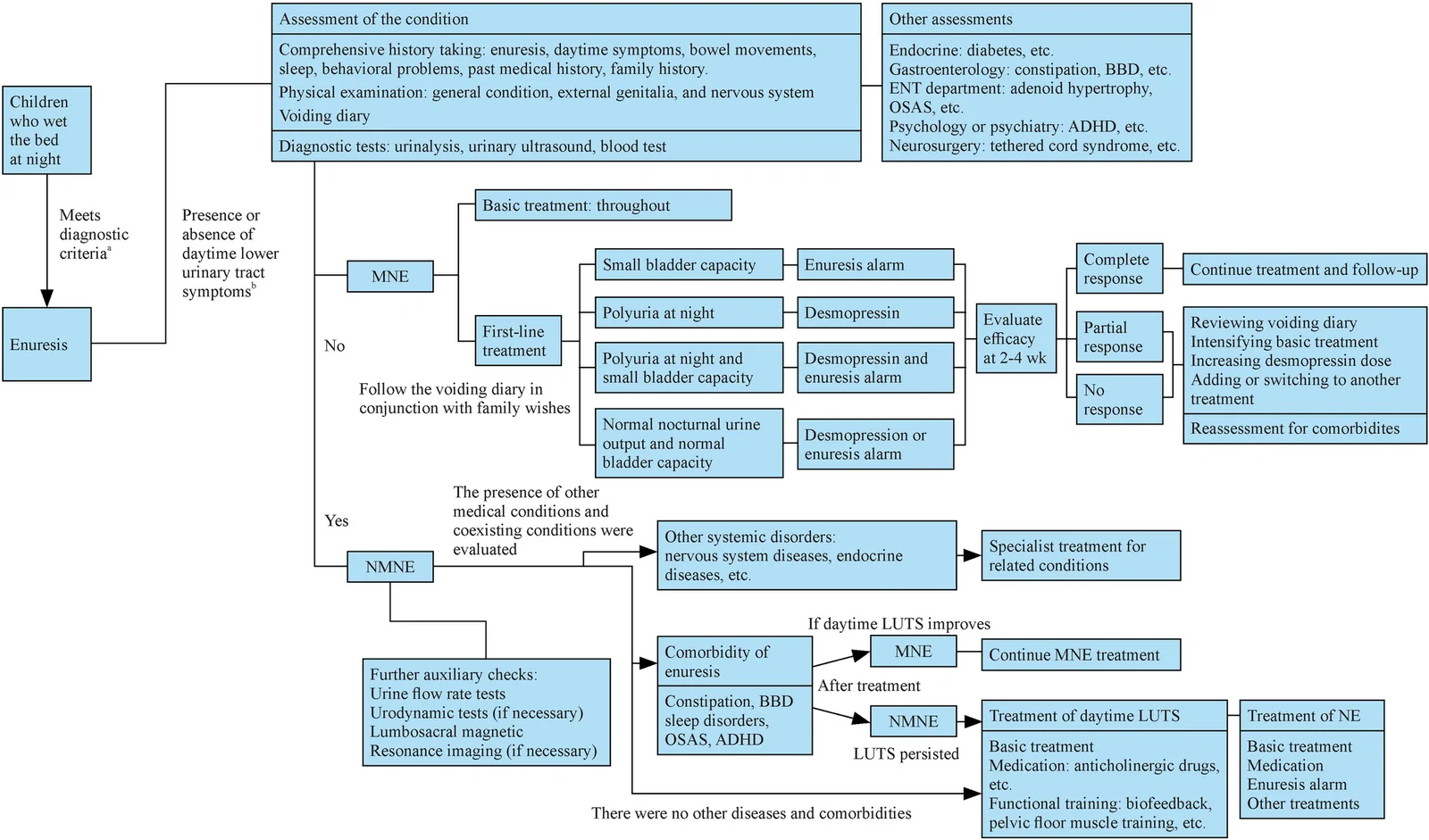
Management pathway for children with nocturnal enuresis. **a** Diagnostic criteria: age older than five years old; the child urinates involuntarily during sleep at least once a month for more than three months.** b** Daytime lower urinary tract symptoms: daytime incontinence, increased voiding frequency, urgency, decreased voiding frequency, an intermittent stream, hesitancy, or voiding difficulty (abdominal pressure required to promote urination initiation). *BBD*, bladder and bowel dysfunction, *ENT*, department of ear, nose, and throat, *OSAS*, obstructive sleep apnea syndrome, *ADHD*, attention deficit hyperactivity disorder, *NE*, nocturnal enuresis, *MNE*, monosymptomatic enuresis, *NMNE*, nonmonosymptomatic enuresis, *LUTS*, lower urinary tract symptoms, *wk*, week, *etc.*, et cetera. Note: reprinted or reproduced from Journal of Clinical Pediatrics, 2025, Volume 43, Issue 7, 2025, pages 483–499 (https://doi.org/10.12372/jcp.2025.25e0564); under the title “Chinese expert consensus on the management of nocturnal enuresis in children (2025)” by Chinese cooperative group for the management of pediatric enuresis, pediatric nephrology committee of Chinese Medical Doctor Association with permitted copyright

The diagnosis and evaluation of enuresis require a detailed history, a systematic physical examination, and appropriate laboratory and imaging investigations based on the findings. Maintaining a voiding diary is essential during evaluation to guide the clinical diagnosis. A comprehensive diagnostic and management pathway is shown in Fig. 1.

#### History taking

A comprehensive medical history aids the exclusion of underlying diseases, the identification of causes and is crucial for the diagnosis and treatment of enuresis. The history should focus on the following aspects:

**Overall health status:** Assess whether the child’s growth and development are normal. If presentations of chronic kidney disease (e.g., fatigue, poor appetite, growth retardation) appear, check serum creatinine and urine glucose, and perform a thorough physical examination. If the child has excessive thirst with the need to drink water at night, polyuria and polydipsia, test urine glucose and blood glucose, renal function, electrolytes, and morning urine osmolality. Evaluate fluid intake and conduct a physical examination to rule out diabetes or other endocrine disorders and renal tubular diseases.

**Nocturnal enuresis pattern:** Determine the frequency of enuresis (nights per week), the number of bedwetting episodes per night, approximate timing, and estimated volume of urine per void. Inquire about the child’s arousal during NE to assess the “voiding urge-awakening” function. The arousal response to bladder fullness can be categorized into five levels, from weakest to strongest: failure to awaken after urination; awakening immediately after urination; awakening when more than half of urination; awakening at the start of urination, and awakening upon the initial feeling of urinary urgency [15].

**Daytime voiding habits:** Inquire about daytime incontinence, increased voiding frequency, urgency, voiding difficulty, delayed initiation of voiding, special holding postures, interrupted stream, or intermittent voiding. These questions screen for possible bladder dysfunction, including overactive bladder (OAB) and voiding dysfunction. Physical examination, along with an assessment of the urinary flow rate and post-void residual urine, is needed. Urodynamic evaluation may be necessary.

**Bowel habits:** Assess for symptoms of constipation, as this often accompanies NMNE. According to the Rome IV criteria [16], constipation can be diagnosed if at least two of the following occur at least once per week for a minimum of one month in a child over four years old: (1) less than two defecations per week; (2) at least one episode of fecal incontinence per week; (3) history of retentive posturing or excessive stool retention; (4) history of painful or difficult bowel movements; (5) the presence of a large fecal mass in the rectum (detected by ultrasound); and (6) history of passing large-diameter stools that may clog the toilet.

**Sleep problems:** Inquire about sleep duration, difficulty falling asleep, difficulty awakening, frequent nightmares, or daytime somnolence. Ask about persistent heavy snoring or episodes of apnea during sleep. If necessary, use relevant questionnaires or scales to assess sleep quality. Be alert for obstructive sleep apnea syndrome (OSAS). If suspected, consider referral to an otolaryngologist.

**Behavioral issues:** Assess the child’s attention and social interaction at home and school. If signs such as progressively worsening attention span, learning difficulties, social withdrawal, or violent tendencies are present, timely consultation with a developmental-behavioral specialist or child psychologist is warranted.

**Medical history:** Inquire about a history of urinary system diseases, neurological disorders, endocrine diseases, or spinal cord or urinary tract surgeries. Document any prior treatments for enuresis and their outcomes.

**Family history:** Note any family history of enuresis in the parents, siblings, or other relatives.

In clinical practice, a structured history questionnaire (Supplemental Table 1 [1]) can be utilized to efficiently gather information about the child’s nocturnal enuresis, daytime voiding symptoms, and any other potential underlying conditions. LUTS can be identified using the diagnostic questions in Table 2 [17]; an affirmative answer to any question in the table suggests the possibility of NMNE. In addition, collecting information on conditions that may coexist with enuresis to identify comorbidities and associated diseases, such as recurrent urinary tract infections, constipation, bladder and bowel dysfunction (BBD), OSAS, attention deficit hyperactivity disorder (ADHD), and other psychological disorders.

**Table 2:** Diagnostic questions to identify lower urinary tract symptoms

Diagnostic questions
Leakage of urine during the day:
Drops of urine in the underpants
- before voiding
- after voiding
Very wet underpants
Frequency of leakage (episodes/d)
Intermittent or continuous leakage every day
History of daytime incontinence over 3.5 y of age
Urinary frequency (≥ 8 times/d)
Reduced daytime voiding frequency (< 4 times/d)
Sudden and strong urge to urinate
Special holding postures (e.g., leg crossing, heel pressing perineum)
Need to press abdomen in order to urinate (strained abdominal muscles to pass urine)
Interrupted urine stream or several voids one after the other
History of urinary tract infection
Disease or malformation:
Urinary tract malformations
Spinal/neurological diseases
Constipation

Reprinted or reproduced from *British Journal of General practice,* 2017,67(660):328–9. https://doi.org/10.3399/bjgp17X691337 under the title “Enuresis: practical guidelines for primary care” by Vande Walle J, Rittig S, Tekgül S, Austin P, Yang SS, Lopez PJ, et al. (references 17). This is an open access article distributed under the terms of the Creative Commons Attribution 4.0 (CC BY 4.0) license

**Recommendation 3:** Diagnosing NE requires a detailed history, covering overall health status, voiding and bowel habits, sleep and behavioral issues, and past diagnostic and treatment history, while taking care to distinguish other diseases and comorbid conditions (fully agreed: 100%).

#### Physical examination

Children with enuresis should undergo a routine physical examination. This includes assessments of growth and development, with measurements of height, weight, and blood pressure. The genitourinary system should be examined: in boys, check for epispadias (which can be associated with the location of the urethral meatus; 40% of patients have incontinence due to bladder neck insufficiency) and for phimosis. In girls, check for labial adhesions (which can trap urine in the vagina, leading to wet underwear after voiding and giving a false appearance of incontinence). If a girl has continuous dribbling incontinence, an ectopic ureter should be suspected. Ectopic ureter usually arises from the upper pole of a duplex kidney and opens distal to the external urethral sphincter, typically into the urethra or vagina [18].

Neurological examination should focus on detecting spinal developmental anomalies, such as asymmetry of the back, legs, or gluteal cleft. Inspect the lumbosacral region for abnormalities (e.g., sacral dimples, tufts of hair, masses) to rule out tethered cord syndrome. Additionally, perform a targeted neurological examination (e.g., assessing sensory function and gait), observing the gait, lower limb muscle strength, muscle tone, and pathological reflexes. Examine the feet for deformities (e.g., clubbing of toes, tight Achilles tendons, or toe deformities, which may be seen in cases of neurogenic bladder). Abdominal palpation aids detection of fecal retention, and auscultation of bowel sounds can assist in assessing bowel function.

**Recommendation 4:** The diagnostic workup for NE requires a routine physical examination, assessing overall health and checking for any genitourinary anomalies, spinal developmental abnormalities, or neurological signs in the lower limbs. (fully agreed: 97.5%).

#### Ancillary investigations

For all newly diagnosed children, performing a routine urinalysis, including urine glucose, leukocytes, erythrocytes, protein, specific gravity, and osmolality, is recommended to help distinguish diabetes, urinary tract infection, and other kidney diseases. Further tests should be performed if necessary. If kidney or other systemic diseases are suspected, liver and renal function tests and other relevant blood tests should be performed.

Based on the history and physical examination, for children suspected with an underlying urogenital anomaly or those who have not previously undergone urinary tract ultrasound, this is recommended and, if necessary, further magnetic resonance imaging (MRI) [18]. Urinary ultrasound should include both kidneys, ureters, and bladder (measuring maximum bladder capacity and post-void residual volume), which can detect structural abnormalities of the urinary tract, increased bladder wall thickness, or retained residual urine. If undetected constipation is suspected, abdominal and pelvic ultrasound can be performed, and, if needed, a rectal examination and abdominal X-ray can be performed [16]. If a neurological disorder is suspected, a lumbosacral MRI should be performed to evaluate for spinal cord or neurological abnormalities. If there is a history of urethral or bladder surgery or a presentation with straining during urination, interrupted flow, abnormal urinary stream, or prolonged voiding time, consider urodynamic studies, especially when there are signs of NMNE. For children with significant abnormal daytime voiding symptoms, particularly those with OAB, repeated urodynamic studies or uroflowmetry combined with electromyography can be used to assess dysfunctional voiding. If other urinary or systemic disorders are suspected (e.g., structural anomalies of the urinary tract, diabetes insipidus, diabetes mellitus, adenoid hypertrophy, BBD, OSAS, ADHD, or tethered cord syndrome), refer to relevant specialists (pediatric urology, endocrinology, ENT, gastroenterology, neurosurgery, or psychology). For OAB potentially triggered by constipation, if daytime symptoms persist after constipation has improved, urodynamic evaluation is also needed.

**Recommendation 5:** Ancillary investigations are important for NE diagnosis and evaluation. It is recommended that newly diagnosed children undergo urinalysis and ultrasound of the urinary system. For those with significant daytime LUTS and/or abnormal bowel habits, consider urodynamic studies and lumbosacral MRI (fully agreed: 100%).

#### Voiding diary

A voiding diary should be kept, recording at least two days (not necessarily consecutive) of daytime records and seven consecutive nights, detailing fluid intake and voiding events. This diary reflects the child’s daytime fluid intake, voiding habits, and the occurrence of nocturnal enuresis. Using diary data, clinicians can assess bladder capacity via maximum voided volume (MVV) and total nocturnal urine output via total voided volume (TVV). This allows determination of the subtype of enuresis (nocturnal polyuria, small bladder capacity, combined nocturnal polyuria with small bladder capacity, or normal nocturnal urine volume with normal bladder capacity) and guides treatment. The voiding diary should accurately reflect the child’s voiding pattern. The daytime diary should record the times and volumes of all fluid intake and urination events from waking to bedtime to evaluate voiding frequency and MVV (Table 1). The nighttime diary should record the volumes of any nighttime voids (if applicable), diaper weight gain, and the first morning void to assess TVV (Table 1). Bowel movements should also be noted [5, 6, 8, 9]. Refer to sample voiding diaries (Supplemental Tables 2 and 3) and the normal reference values for MVV and TVV [19] (Table 3). During recording, it is recommended that the child have adequate fluid intake during the day, restrict fluids two hours before bedtime, and empty his or her bladder before going to sleep. Prior to starting the diary, clinicians should fully communicate with the parents and child, and provide detailed explanations on how to correctly record the diary to ensure data accuracy and reliability. This facilitates a precise assessment of the condition and provides a basis for subsequent treatment.

**Table 3:** Expected age-related bladder capacity and interpretation of maximum and total voided volume at night

Age (y)	EBC (mL)	Small bladder capacity: MVV (mL)^a^ < 65% of EBC	Nocturnal polyuria: TVV (mL)^b^ > 130% EBC
5	180	117	234
6	210	137	273
7	240	156	312
8	270	176	351
9	300	195	390
10	330	215	429
11	360	234	468
12–18	390	254	507

^a^MVV is measured (excluding the first morning void) for at least 2d; weekends or holidays are ideal periods. Any daytime urinary leakage or fluid intake should be recorded

^b^TVV is determined by summing the volume of the first morning void and nighttime urine output (including diaper weight gain) to calculate nocturnal urine production

*EBC* expected bladder capacity, *MVV* maximum voided volume, *TVV* total voided volume

Reprinted or reproduced from *European Journal of Pediatrics*, 2012;171:971–83. http://doi.org/10.1007/s00431-012-1687-7 under the title “Practical consensus guidelines for the management of enuresis” by Vande Walle J, Rittig S, Bauer S, Eggert P, Marschall-Kehrel D, Tekgul S (references 19). This is an open access article distributed under the terms of the Creative Commons Attribution 2.0 (CC BY 2.0) license

It is recommended to keep a voiding diary during the initial evaluation. For children on medication, parents are encouraged to bring a completed voiding diary at follow-up visits allowing clinicians to assess the medication’s efficacy. In particular, if the child is not yet fully dry at night, keeping another detailed voiding diary is recommended to evaluate efficacy and adjust management. Encourage children to participate in recording their diary and provide rewards and positive feedback to improve adherence while also fostering self-awareness and self-management skills. Some studies suggest that shortening the recording period may improve compliance. A modified diary protocol consisting of three weekend nights and two daytime recordings with standardized fluid intake can increase adherence [20]. This modified approach can be used for some patients during initial or follow-up evaluations.

**Recommendation 6:** Children with NE keep a voiding diary to guide clinical diagnosis and to evaluate treatment efficacy (fully agreed: 95%).

### Management of nocturnal enuresis

#### Age to initiate treatment

Many international guidelines recommend initiating treatment for enuresis at approximately 5–6 years of age. However, the decision to treat is not solely age-dependent; it should be based on the burden of symptoms on the child and their family. Enuresis adversely affects health-related quality of life (HRQoL) of children. In particular these impacts self-esteem, mood, and interpersonal relationships [21], especially if symptoms persist into adulthood [22]. Therefore, a “wait-and-see” approach is inappropriate for children who are already distressed by enuresis. Different children and their parents have varying tolerances for bedwetting and experience different degrees of distress caused by the condition. An individualized assessment should consider the severity of the condition and the desire for treatment. If bedwetting causes significant distress to the child and family, treatment should be initiated as early as possible. Generally, treatment is recommended for children over five years of age. For children under five years of age whose bedwetting causes significant distress to the child or family, consultation and basic evaluation (including history, physical exam, and urinalysis) are recommended to rule out underlying conditions and to provide counseling and basic management advice. The goal of treatment is to improve symptoms, reduce the frequency of bedwetting, and alleviate the impact of enuresis on the child’s life, family life, and social adaptation. Treatment efficacy categories (compared to baseline wet nights in the month) are: complete response (100% reduction), partial response (50%–99% reduction), and no response (< 50% reduction) [4].

**Recommendation 7:** The age to initiate treatment for childhood enuresis should be individually assessed; generally, treatment is recommended for children over five years old. Children with NMNE often require more aggressive evaluation and multidisciplinary management (fully agreed: 100%).

#### Basic treatment

Clinicians should educate children with NE along with their parents, providing basic information about the condition. It is important to emphasize that enuresis is not the child’s fault and to discourage punishment or blame. Guidance on a healthy lifestyle is the foundation of treatment for pediatric enuresis. Some children can improve with basic interventions, such as adjustments in lifestyle, habits and behavioral training alone. Basic treatment should be maintained throughout the entire management process. For younger children or those with minimal impact on daily life, a 1–3 months trial of basic therapy can be attempted.

#### Adjusting daily routine:

Establish a regular schedule. Encourage the child to maintain normal fluid intake during the day, ensuring adequate daily fluids (Table 4) [23]. Advise avoiding foods or beverages containing theophylline or caffeine. Dinner should be early, light, and low in salt and fat. The child should avoid vigorous activity or excessive excitement after dinner. Ensure the child goes to bed early and has a regular sleep schedule, which improves nighttime sleep quality and ensures adequate sleep duration. The child should not eat within the 2–3 hours before bedtime; fluid intake should be restricted two hours before bedtime, and consumption of foods with high water content (such as porridge, soups, milk, fruits, and juice) in the late evening should be avoided.

**Table 4:** Suggested daily intake of drinks for children and adolescents

Age (y)	Suggested daily intake of drinks (mL)	
	Girls	Boys
4–8	1000–1400	1000–1400
9–13	1200–2100	1400–2300
14–18	1400–2500	2100–3200

Reprinted or reproduced from *Annals of Indian Psychiatry,* 6(1):4–14, 2022. https://doi.org/10.4103/aip.aip_102_21; under the title “A clinical review of enuresis and its associated psychiatric comorbidities” by Pole R, Vankar GK, Ghogare AS. This article is available under the terms of the Creative Commons Attribution-NonCommercial-ShareAlike License (CC BY-NC-SA), which permits non-commercial use, distribution and reproduction in any medium, provided the original work is properly cited

#### Develop good voiding and bowel habits

Establish a habit of regular daytime urination (4–7 times per day) and ensure that the child voids before going to bed. In addition, it is recommended that the child consume a fiber-rich diet and develop a regular bowel movement habit. For children with constipation, active treatment should be conducted in parallel with enuresis management.

#### Positive reinforcement and reward system

With physician help, parents should build confidence in overcoming enuresis, and continually reinforce positive behaviors and motivation. Parents should not blame the child but instead provide frequent encouragement to reduce the psychological burden of the condition on the child. Actively involve the child in the treatment process and offer appropriate rewards.

#### Keep a voiding diary

Instruct parents to diligently maintain a voiding diary, which aids in the assessment of enuresis severity and provides individualized guidance for treatment. If the child has daytime symptoms, filling out the voiding diary can be part of their therapy. In some cases, simply keeping a voiding diary for one week has helped children develop good voiding and bowel habits, leading to a significant improvement in enuresis.

**Recommendation 8:** For younger children or those whose enuresis has a minimal impact on daily life, basic treatment can be attempted first; basic treatment measures should be maintained throughout the entire course of enuresis management (fully agreed: 97.5%).

#### Management of monosymptomatic enuresis

##### First-line treatments

Desmopressin and enuresis alarms are established as first-line treatments for MNE in numerous international guidelines [6, 8] with the potential to achieve complete resolution in the majority of cases. Desmopressin, a synthetic analog of vasopressin, reduces nocturnal urine production facilitating its containment within the functional bladder capacity. Clinical studies show that desmopressin leads to a ≥ 50% reduction in wet nights in 50%–85% of patients, with 40%–70% achieving near-complete dryness during therapy [24]. The enuresis alarm incorporates a moisture sensor that activates upon detecting urine, awakening the child who should then complete voiding in the toilet. This conditions the arousal response to bladder fullness, with the goal of the child eventually awakening spontaneously to the sensation of a full bladder. The enuresis alarm has an efficacy rate as high as 50%–70% [6], and its effectiveness is closely related to the motivation and cooperation of the child’s family [25]. Studies have shown that for children who do not respond to alarm therapy alone, combining an enuresis alarm with desmopressin can still achieve a complete response in approximately 74% of patients [26]. The choice between first-line treatments should be individualized, considering the enuresis subtype and crucially, the preferences and motivation of the child and family. If the initially chosen treatment (desmopressin or alarm) does not achieve dryness, the situation should be reevaluated, and an alternative or an additional modality should be selected [27].

For children with nocturnal polyuria, desmopressin is the first choice. The recommended initial dose of oral desmopressin (tablet) for the first course is 0.2 mg once nightly (q.n.). The treatment effect should be evaluated every 2–4 weeks. For children with a partial or no response, the dose of desmopressin may be increased incrementally by 0.1–0.2 mg every 2–4 weeks [10, 28], up to a maximum of 0.6 mg q.n. A fast-melting oral lyophilisate has superior bioavailability and efficacy over the oral tablet, with an additional advantage of not requiring water for administration. However, the melt is not yet commercially available in China. For reference, a 0.2 mg desmopressin oral tablet is bioequivalent to a 120 µg desmopressin melt. During the treatment process, simultaneous assessment of medication adherence, lifestyle, and fruid intake habits is recommended. Moreover, ancillary tests should be conducted if necessary to identify any comorbid conditions and rule out issues such as noncompliance. Additionally, voiding diaries should be reviewed to determine if dose adjustment is needed.

**Fig. 2:**
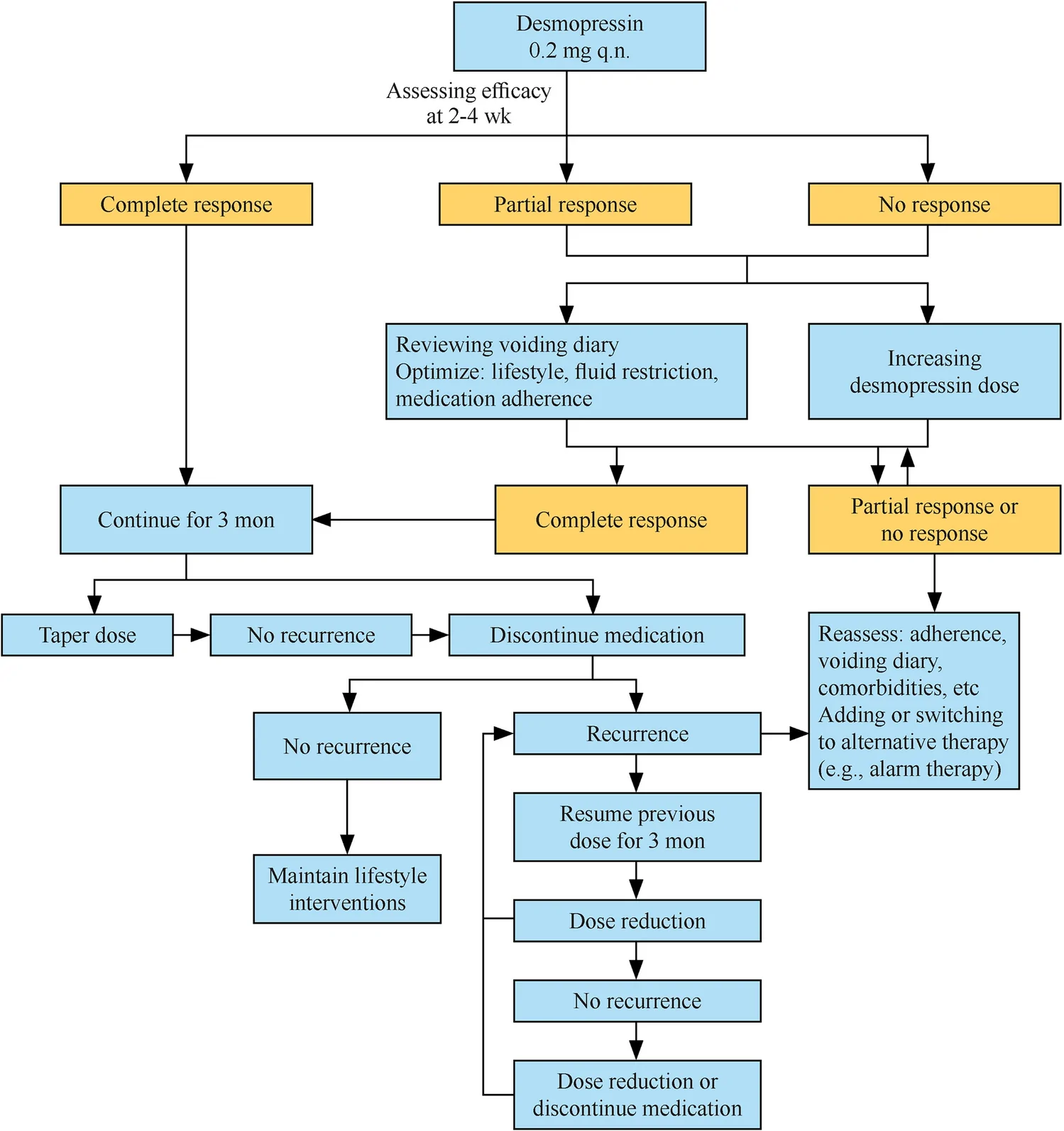
Follow-up and withdrawal strategies for desmopressin treatment in children with nocturnal enuresis. *q.n.*, every night; *wk*, week;* e.g.*, for example; *etc.*, et cetera. Note: reprinted or reproduced from *Journal of Clinical Pediatrics,* 2025, Volume 43, Issue 7, 2025, pages 483–499 (https://doi.org/10.12372/jcp.2025.25e0564); under the title “Chinese expert consensus on the management of nocturnal enuresis in children (2025)” by Chinese cooperative group for the management of pediatric enuresis, pediatric nephrology committee of Chinese Medical Doctor Association, with permitted copyright

Following a three months period of successful therapy (complete response) at a stable dose, discontinuation may be attempted. This can be done either abruptly or via a structured taper. If the child remains dry after stopping or tapering, then the drug can be discontinued. If enuresis recurs after discontinuation, it is important to reevaluate potential causes. The child can then receive further desmopressin treatment, and after at least another three months of maintenance therapy, a structured withdrawal can be considered. This is gradually reducing the dose until discontinuation, rather than stopping abruptly, to reduce the risk of a further relapse [29–33]. Additionally, compared with time interval-based withdrawal (increasing the interval between doses), dose-reduction-based withdrawal (lowering the dose) is more effective in reducing relapse [30, 34]. Currently, there is significant heterogeneity in clinical practice regarding the optimal structured withdrawal strategy. Both the rate and duration of tapering lack high-quality evidence, and international guidelines have not reached a unified recommendation. Some literature reports suggest first reducing the dose by half and then discontinuing after 2–4 weeks [10, 30]. If no bedwetting occurs after dose reduction, continue tapering until cessation; if enuresis recurs during dose reduction, revert to the previous effective dose and maintain for another three months (Fig. 2).

The main adverse effects of desmopressin are hyponatremia and water intoxication, although these effects are extremely rare. Severe hyponatremia can present with symptoms, such as headache, nausea, and vomiting. In clinical trials for primary enuresis, the overall incidence of adverse events with desmopressin was low [35]. Studies evaluating the long-term efficacy (≥ 6 months) and safety of oral desmopressin indicate it is well tolerated and effective in the long term [36] and does not significantly affect blood pressure, laboratory parameters (including serum sodium, potassium, and osmolality) or urinalysis results [24]. In healthy children, the risk of hyponatremia and water intoxication from desmopressin is extremely low; therefore, routine monitoring of serum sodium is not necessary [37]. Hyponatremia can occur only if the antidiuretic effect of desmopressin coincides with excessive fluid intake and/or prolonged drug bioactivity [38]. Therefore, when prescribing desmopressin, other medications that may increase the risk of hyponatremia should be avoided. Additionally, the child and family should be educated on the importance of restricting nighttime fluid intake. Increasing the dose of desmopressin may increase the risk of hyponatremia. Studies also suggest monitoring serum sodium when the dose is increased, although there is no strong evidence specifying the dose at which this monitoring becomes necessary [37]. We suggest checking the serum sodium concentration within seven days when the dose is increased to 0.4 mg q.n. or above [37]. Moreover, strengthen family education to ensure that they do not adjust the medication dose independently. Research has shown that when desmopressin doses up to 0.6 mg q.n. are used, it is well tolerated by patients [39].

Precautions for taking desmopressin include: (1) taking the medication one hour before bedtime, with a small amount of water [6]; (2) restricting fluid intake for two hours before bedtime and eight hours after taking the medication to ensure therapeutic effect and avoid adverse reactions; (3) if the child develops a fever requiring significant fluid supplementation, desmopressin should be temporarily stopped to prevent water intoxication. If the medication has already been taken, fluid intake should still be restricted; (4) monitoring blood pressure and serum sodium when necessary. If the child shows no response after 6–8 weeks of treatment, consider combining treatment with the enuresis alarm, or using other treatment methods or combination therapy (see “second-line treatment”). It is recommended that primary health-care institutions refer the child to a specialized enuresis clinic for further management.

For children with a normal nighttime urine volume but reduced bladder capacity, an enuresis alarm is the first choice. The alarm should be used every night, typically for eight consecutive weeks or longer, until the child has no bedwetting episodes for 14 consecutive nights. Efficacy is closely related to the enthusiasm and cooperation of the child’s family. Since alarm therapy can disrupt the sleep of both the child and the parents and often takes a long time to show effectiveness, it is necessary for physicians to establish good communications and ensure realistic expectations for the child and family prior to initiation. If the parents have difficulty in cooperating, other treatment options can be considered. Proper training and guidance are the keys to success; during the implementation process, the frequency of bedwetting should be monitored, and psychological positive reinforcement techniques should be continuously used to enhance treatment motivation of the family, while establishing a comprehensive follow-up plan until treatment success. An enuresis alarm can also be used during the dose reduction phase of desmopressin therapy to promote the spontaneous awakening of the child and reduce the chance of relapse. Studies have shown that wireless alarms have a higher complete response rate than wired alarms [40]. Precautions for alarm therapy include the following: (1) parents should be mentally prepared: when the underwear or bedding becomes wet and triggers the alarm, if the child does not wake up, the parent should wake the child and help the child get up to finish voiding; (2) the child should use the alarm every night; (3) if there is no improvement after six weeks of treatment, the therapy should be discontinued, and (4) if there are signs of improvement after treatment (e.g., smaller wet spots, occasional dry nights), continue the therapy until the child has no bedwetting episodes for 14 consecutive nights. Alarm therapy requires a high level of compliance. Frequent alarm activations may affect sleep and impose a burden on the family; therefore, alarm use is recommended only when the family can actively cooperate. Efficacy is closely related to the motivation and cooperation of the child and family. Predictive factors for successful outcomes include high motivation in the child, strong family support, ability to tolerate sleep disruption during the initial phase, and the absence of significant comorbidities such as ADHD or severe psychosocial stressors. Alarm use is recommended primarily when the family can actively and consistently cooperate.

For children with both nocturnal polyuria and small bladder capacity, either desmopressin or an enuresis alarm can be used. However, literature reports indicate that combining desmopressin with an enuresis alarm is more effective than using desmopressin or an alarm alone [26, 41, 42].

For enuretic children with a significantly small bladder capacity (experiencing more than one bedwetting episode per night), those who have failed alarm therapy, or those who show no improvement even after combination therapy, a comprehensive evaluation of the children’s condition should be conducted. Treatment options may include desmopressin, anticholinergic medications, bladder function training, psychological-behavioral therapy, or a combination of these. Effective measures for bladder function and behavioral training include: cultivating healthy lifestyles and dietary habits, scheduled voiding every two or three hours to avoid urinary retention, adopting a proper posture during voiding, and encouraging good habits through reward systems. Current evidence indicates that anticholinergic medication combined with desmopressin is superior to desmopressin alone or alarm therapy for MNE, with good safety [41–46]. The possible mechanism is that in some enuretic children without daytime LUTS, nocturnal detrusor overactivity limits bladder capacity; anticholinergic drugs can reduce detrusor overactivity and therefore increase bladder capacity.

**Recommendation 9:** Desmopressin and enuresis alarms are first-line treatments for MNE. Desmopressin is preferred for children with nocturnal polyuria, whereas the enuresis alarm is recommended for those with small bladder capacity. Combined therapy is advised for children with both conditions. The final treatment plan should be individualized based on the clinical profile and the preferences of the parents and child (fully agreed: 100%).

**Recommendation 10:** The recommended initial dosage of desmopressin is 0.2 mg once nightly, which may be titrated up to a maximum of 0.6 mg once nightly based on response. Although hyponatremia and water intoxication are potential adverse effects, the risk is extremely low with correct adherence to fluid restriction, and the drug is generally well tolerated. A treatment course of three months is typical before attempting discontinuation in responders, which can be abrupt or via a graded taper (fully agreed: 100%).

#### Second-line treatment

In children with MNE who do not respond to the first-line therapy, a thorough reevaluation is mandatory prior to considering second-line options. This includes assessing treatment adherence, lifestyle factors, and fluid intake habits, using a voiding diary, and excluding comorbidities. Following this a trial of anticholinergic medications may be considered. Anticholinergic drugs can effectively reduce the frequency of nocturnal bedwetting. If a child has an excessive nighttime voiding frequency, a small functional bladder capacity, or suspected bladder overactivity (after excluding neurogenic bladder or other organic diseases), a combination of an anticholinergic medication and desmopressin may be considered [46]. Oxybutynin is commonly used. The recommended dosage for the immediate-release formulation is 2.5 mg twice or three times daily (which can be increased to a maximum of 5.0 mg three times daily). For the extended-release formulation, the recommended starting dosage is 2.5–5.0 mg once daily (which can be increased to 10.0 mg once daily in older children). Recent literature supports the use of other medications, such as tolterodine, solifenacin, and propiverine, in primary monosymptomatic nocturnal enuresis (PMNE) [47, 48] to improve bladder function or detrusor overactivity, although these medications currently lack pediatric indications. The recommended dose of tolterodine is 1.0–2.0 mg twice daily, not exceeding 4.0 mg/day; the recommended starting dose of solifenacin is 2.5–5.0 mg once daily, up to a maximum of 10.0 mg/day; and the recommended starting dose of propiverine is 0.8 mg/kg/day, up to a maximum of 1.2 mg/kg/day. For children < 35 kg, propiverine is suggested in a twice-daily regimen; for those ≥ 35 kg, dosing starts at 30 mg once daily and does not exceed 45 mg/day [49, 50]. The most common adverse effects in pediatric practice include constipation (which can exacerbate lower urinary tract dysfunction), increased post-void residual volume (raising the risk of urinary tract infection), and dry mouth (potentially contributing to dental caries) [51]. Of note, oxybutynin has been associated with mood fluctuations and other neuropsychiatric side effects [52]. These medications must be prescribed under specialist supervision, with particular attention to bowel habits and periodic assessment of post-void residual urine volume. Tricyclic antidepressants (e.g., imipramine) have documented efficacy but are rarely used in current practice due to their side-effect profile and the availability of safer alternatives; they are not recommended in this consensus.

**Recommendation 11:** For children with MNE who do not respond to the first-line therapy (correct use of an enuresis alarm or adequate dosing of desmopressin), other treatment methods or combination therapies should be selected, such as combining desmopressin with an anticholinergic (fully agreed: 100%).

#### Management of non-monosymptomatic nocturnal enuresis

##### Treatment principles

Management of NMNE begins with a comprehensive evaluation, with particular emphasis on characterizing LUTS and identifying comorbidities, especially constipation. Essential investigations include maintaining a voiding diary and, in many cases, performing urodynamic studies. Ultrasound measurement of post-void residual urine combined with uroflowmetry is a standard initial approach to screen for lower urinary tract dysfunction. Basic treatment should be implemented throughout the entire process (section “Basic treatment”). In children with NMNE, standard urotherapy is emphasized, including health education and encouragement of behavioral and lifestyle modifications (Table 5) [53]. The management of daytime LUTS should be prioritized over the treatment of nocturnal enuresis, except in cases where they are minimal (e.g., only mild changes in voiding frequency) [6, 27]. If constipation is present, improving the child’s bowel habits while treating nocturnal enuresis is crucial for improving voiding ability. Moreover, attention should be given to treating other associated complications and comorbid conditions.

**Table 5:** Urotherapy recommendations for patients with lower urinary tract symptoms

Recommendations
Correcting daily fluid intake
Avoiding fluid intake 2 h before bedtime and at night
Avoiding carbonated, caffeine-rich drinks
Eating regular meals throughout the day
Consuming a diet with correct amounts of protein and salt
Avoiding constipation, if necessary, seek pharmacological treatment for constipation
Micturating at regular 2–3 h intervals
Avoiding urine retention in the bladder
Maintaining appropriate posture during urination, straightening the torso during micturition. The height of the toilet should be adapted to the child to enable proper support of the feet

Reprinted or reproduced from *Clinical Medicine Insights: Pediatrics*, 2020,14: https://doi.org/10.1177/1179556520975035 under the title “Voiding disorders in pediatrician’s practice” by Rakowska-Silska M, Jobs K, Paturej A, Kalicki B (references 53). This is an open access article distributed under the terms of the Creative Commons Attribution 4.0 (CC BY 4.0) license

If daytime symptoms persist after the improvement of associated symptoms, additional treatment modalities, including both pharmacological and non-pharmacological therapies, should be introduced. Specifically, these include pharmacological options (e.g., anticholinergic medications, β3-adrenergic receptor agonists) and non-pharmacological treatments (e.g., specific urotherapy techniques, various forms of pelvic floor muscle training, biofeedback, transcutaneous electrical nerve stimulation, psychological-behavioral therapy). If daytime symptoms improve but nocturnal enuresis symptoms persist, standard therapy for nocturnal enuresis, as used for MNE, should be initiated. If there are no improvements in either daytime or nighttime symptoms, strategies may include dose escalation, addition of another medication (e.g., combining desmopressin with an anticholinergic), or combination of pharmacotherapy with other modalities (e.g., nerve stimulation, biofeedback). These approaches still focus primarily on treating daytime symptoms.

**Recommendation 12:** The initial management of NMNE should prioritize the treatment of daytime symptoms, with a focus on treating LUTS and comorbid conditions. Nocturnal enuresis symptoms that persist after the resolution of daytime symptoms should be managed according to the principles for MNE (fully agreed: 100%).

#### Pharmacological management for daytime symptoms

Anticholinergic medications act primarily by antagonizing muscarinic receptors in the detrusor muscle, thereby reducing involuntary contractions and increasing functional bladder capacity. They are indicated for children with NMNE, particularly those with symptoms of OAB or detrusor overactivity. The use of anticholinergic therapy in these children can lead to an increase in functional bladder capacity and improved urinary control [18]. Commonly used anticholinergic drugs include oxybutynin, tolterodine, solifenacin, and propiverine (for specific usage indications, refer to section “second-line treatment”). Evidence suggests that tolterodine may modestly reduce episodes of urge incontinence, while solifenacin can increase bladder capacity but may not significantly improve incontinence frequency compared to placebo. Propiverine has been shown to increase voided volume and reduce urinary frequency [54]. Anticholinergic medications should be prescribed under specialist supervision. Prior to initiation, neurogenic bladder and anatomical abnormalities must be excluded. During treatment, patients should be monitored for adverse effects, such as dry mouth, blurred vision, and constipation, and post-void residual urine volume, should be assessed regularly. β3-adrenergic receptor agonists, commonly used in adults with OAB, promote detrusor relaxation and increase bladder capacity. Their efficacy is comparable to anticholinergics, but they have a different side-effect profile, as they do not typically cause anticholinergic adverse events such as dry mouth or constipation, nor do they impair voiding contraction [55]. Recent evidence supports the use of selective β3 agonists, such as mirabegron and vibegron [56, 57], for the management of OAB in pediatric populations. Consultation with a pediatric nephrologist is recommended when mirabegron is considered. Blood pressure and post-void residual urine volume should be monitored before and during treatment, and vigilance for potential germ cell toxicity is advised. Further investigations are needed to determine the efficacy and safety of vibegron in children and clarify its potential impact on germ cell toxicity.

**Recommendation 13:** Anticholinergic medications and β3-adrenergic receptor agonists are therapeutic options for patients with NMNE (fully agreed: 100%).

#### Non-pharmacological management

Pelvic floor muscle training (e.g., deep squats, bridge exercises) can strengthen the pelvic floor, gluteal, and abdominal muscles, promoting muscle and ligament strength during childhood. This contributes to improved bladder control and urethral sphincter function [58, 59].

Biofeedback therapy utilizes instruments such as perineal electromyography combined with uroflowmetry to identify voiding-phase dysfunction. It guides children to achieve proper control of the external urethral sphincter during micturition. The reported success rate of biofeedback therapy is about 64% [60].

The mechanism of transcutaneous electrical nerve stimulation (TENS) is not fully elucidated but may involve stimulation of lower abdominal nerves, activating inhibitory sympathetic pathways and suppressing excitatory parasympathetic (pelvic) nerve activity. This may promote central nervous system remodeling and suppress involuntary detrusor contractions [61]. Studies on TENS for enuresis have shown variable outcomes, although it can reduce wetting frequency [62] and may be considered for cases refractory to other treatments [7].

**Recommendation 14:** Pelvic floor muscle training and biofeedback therapy can be considered for children with daytime LUTS (e.g., urgency, urge incontinence, voiding difficulty). Transcutaneous electrical nerve stimulation may be an option for patients with refractory NMNE, particularly when associated with OAB and constipation (fully agreed: 100%).

#### Other

##### Treatment of comorbidities

Studies indicate that 36%–80% of children with enuresis have comorbid constipation [63, 64], and successful management of functional constipation positively influences enuresis outcomes [65, 66]. Constipation increases the risk of enuresis. Pathophysiological mechanisms may include rectal distension causing mechanical compression of the bladder, reducing functional capacity, and inducing detrusor overactivity. Shared neuronal pathways due to the common embryological origin of the bladder and rectum may also contribute to this association [67]. For children with OAB potentially triggered by constipation, dietary and fluid intake guidance is recommended. After relieving constipation, if daytime symptoms still persist, further evaluation is needed; if only nocturnal enuresis remains, then treatment should focus on nocturnal symptoms.

Comorbidities such as ADHD are also common among enuretic children, with prevalence rates of up to 28.3% [68]. When enuresis occurs with ADHD, which is associated with lower response rates to standard enuresis treatment, prompt referral for ADHD evaluation and concurrent management is recommended.

Sleep-disordered breathing is another common comorbidity, 31.4% of affected children have enuresis. One study reported that among children with both conditions, adenoidectomy led to a reduction in the severity of enuresis in 81.1% of cases within one month, with 64.9% achieving complete dryness. An additional 18.9% became dry by the three months follow-up [69]. Therefore, surgical intervention (e.g., adenotonsillectomy) should be considered for children with refractory enuresis and comorbid OSAS due to adeno-tonsillar hypertrophy.

**Recommendation 15:** Children with enuresis who have comorbid conditions (e.g., constipation, ADHD, OSAS) often have a lower response to basic therapy. Concurrent management for these comorbidities is essential (fully agreed: 97.5%).

##### Role of primary health care institutions

Primary health-care institutions (e.g., district or county-level hospitals, community hospitals, maternal and child health centers) with capabilities for basic urinalysis and urinary ultrasound can initiate management of MNE. First-line treatment (desmopressin, enuresis alarm) should be selected based on the voiding diary and clinical subtyping, following individualized discussion with the family (Fig. 1).

Although these treatments are successful for most children, some do not respond. Therefore, referral to a specialized enuresis clinic or hospital is recommended for children who do not respond to first-line therapy or for those diagnosed with or suspected of having NMNE [17]. Recommended evaluations in specialized settings may include uroflowmetry, urodynamic studies, and lumbosacral MRI (Fig. 1). Multidisciplinary consultation may be necessary for complex cases to assess the possible involvement of other organ systems. Establishing efficient referral pathways to regional pediatric enuresis centers is encouraged.

**Recommendation 16:** Primary health-care institutions may initiate management of MNE. Referral to a specialized center for further diagnosis and treatment is recommended if there is no response to first-line treatment or if NMNE is diagnosed or suspected (fully agreed: 100%).

#### Traditional Chinese medicine approaches

Traditional Chinese medicine (TCM) attributes enuresis primarily to kidney qi deficiency. The therapeutic principle is tonification, aimed at warming and strengthening the kidney and calming the mind. Treatment is based on syndrome differentiation: for patterns of kidney qi insufficiency and cold in the lower origin, warming the kidney and securing essence is used; for spleen-lung qi deficiency, tonifying qi and securing essence is applied; and for damp-heat in the liver meridian, clearing heat and draining dampness is used. Modalities include Chinese herbal medicine, acupuncture, therapeutic massage, and medicinal plaster therapy [70, 71]. Some herbal treatments may also help improve sleep arousal. Detailed protocols can be found in the clinical practice guidelines of TCM for pediatric enuresis patients [72].

**Recommendation 17:** Comprehensive TCM therapies, including herbal medicine and external therapies (e.g., acupuncture, massage, medicinal patches), may improve enuresis-related symptoms based on syndrome differentiation. They may be integrated with Western medicine in the treatment of enuresis (fully agreed: 100%).

#### Refractory enuresis

A diagnosis of refractory enuresis should be considered when there is no response (< 50% reduction) after at least three months of standard therapy [73]. In such cases, a systematic reevaluation is mandatory. First, the child’s lifestyle should be reviewed, and adherence to the correct treatment and correctness should be evaluated, including compliance with basic measures (e.g., fluid management), correct medication administration, and proper use of the enuresis alarm; second, the condition should be reassessed: a detailed voiding diary should be recorded to verify the subtype of enuresis and its severity. Follow-up urinalysis, blood tests, and renal bladder ultrasound (with post-void residual urine volume) should be performed to exclude urinary tract structural abnormalities or other systemic diseases. Previously unperformed investigations, such as lumbosacral MRI to exclude neurological abnormalities (e.g., tethered cord syndrome) and urodynamic studies to assess for bladder dysfunction or neurogenic bladder and to elucidate the interaction between bladder and bowel symptoms should be completed [6, 73]. Furthermore, a systematic screening for intervenable comorbidities (e.g., adenoid hypertrophy, BBD, OSAS, ADHD) and other potential causes of secondary enuresis (e.g., urinary tract infection, chronic kidney disease, diabetes mellitus, neurological disorders) is essential.

During the management process, an individualized treatment approach should be adopted, with full respect for the preferences of the family and the child. The child should be actively involved in the treatment process. In cases of non-adherence, such as failure to limit fluid intake while on desmopressin or inadequate awakening of the child when an enuresis alarm is used, family education is essential to correct implementation before reevaluating treatment efficacy. For children who do not respond to first-line therapy, alternative pharmacological options, such as the addition of an anticholinergic agent, may be considered. First-line treatment may also be reattempted after several years. In patients who are refractory to all medication-based therapies, non-pharmacological interventions, including biofeedback, pelvic floor muscle training, or neuro-stimulation, may be attempted. Where feasible, a multidisciplinary collaborative management model is recommended. Emphasis should be placed on the active participation of both the family and the child, along with regular assessments of treatment adherence, to improve overall outcomes in refractory patients.

**Recommendation 18:** Children with refractory enuresis should undergo a comprehensive re-evaluation. This includes assessing treatment adherence, reviewing the voiding diary, and performing appropriate investigations to identify underlying causes and guide treatment adjustment (fully agreed: 97.5%).

## Limitations and future directions

Several limitations should be ackonwledged. This consensus was developed predominantly by pediatric nephrology experts in China, and some recommendations may therefore be influenced by regional clinical practice patterns. Furthermore, despite a rigorous, despite a rigorous evidence-review process, the available pediatric evidence remains limited in several areas, such as the optimal strategy for desmopressin withdrawal and long-term outcomes of combination therapies.

Future research should focus on: (1) conducting well-designed randomized controlled trials in Chinese pediatric populations to compare the efficacy of different treatment sequences, combination regimens, and structured withdrawal protocols; (2) investigating phenotypic and genetic biomarkers that predict treatment response to enable personalized therapy; (3) exploring the long-term psychosocial outcomes and cost-effectiveness of different management strategies; (4) strengthening multidisciplinary collaboration models integrating nephrology, urology, gastroenterology, and psychology for complex cases.

## Conclusions

This expert consensus presents an updated, evidence-based, and practical framework for managing childhood NE, focusing on clinical practice. Key recommendations include: (1) updated diagnosis: the criteria are updated to include children aged five years or above with at least one involuntary void per month for three months, emphasizing classification into MNE or NMNE based on daytime lower urinary tract symptoms, (2) systematic evaluation: a comprehensive assessment is recommended, including detailed history, physical exam, urinalysis, and a voiding diary to determine the phenotype and identify comorbidities. (3) individualized management: basic therapy (education, lifestyle modification) is foundational for all. For MNE, desmopressin is recommended as first-line treatment for children with nocturnal polyuria, while the enuresis alarm is first-line for those with reduced bladder capacity. The choice should be individualized based on the clinical profile and family or patient preferences. For NMNE, management must prioritize daytime symptoms and comorbidities before addressing nighttime wetting. Strategies for partial/non-responders include dose adjustment, combination therapy, and specialized interventions. In summary, by synthesizing evidence and expert experience, this consensus provides 18 specific recommendations to standardize care, promote individualized treatment, and improve outcomes for children with NE.

## Supplementary Information

Below is the link to the electronic supplementary material.

Supplementary file1 (DOCX 38 KB)

## Data Availability

The authors confirm that the data supporting the findings of this study are available within the article and supplementary materials.
