# Supplementary material for: Expert consensus on the management of nocturnal enuresis in children
Source: World J Pediatr. 2026 Jul 10;22(7):709–27. doi: 10.1007/s12519-026-01051-4 (PMC13578046; doi:10.1007/s12519-026-01051-4)
Supplement: Supplementary file 1 [file 12519_2026_1051_MOESM1_ESM.docx]

**Supplemental Table 1 Medical history collection form**

| Item | Response |
| --- | --- |
| Does the child wet the bed during sleep at night | □ Yes □ No |
| 1. Number of bedwetting nights per week | ______ nights/week |
| 2. Number of episodes per night | ______ times/night |
| 3. Approximate time of bedwetting | __________________ |
| 4. Urge-awakening function: (1) not awake after urination, (2) immediately awake, (3) awake after half urination, (4) awake at start, (5) awake upon urge | □ (1) □ (2) □ (3) □ (4) □ (5) |
| 5. Amount of bedwetting per night (measured by diaper weight gain) | ______ g/mL |
| Daytime lower urinary tract symptoms (indicates bladder dysfunction) | |
| 1. Daytime incontinence (suggests bladder overactivity or NMNE) | □ Yes □ No |
| Urine stains before urination (leakage occurring immediately before voluntary voiding) | □ Yes □ No |
| Urine stains after urination (post-void dribbling) | □ Yes □ No |
| Severely wet underwear | □ Yes □ No |
| Frequency of incontinence | ______ times/d |
| Intermittent or continuous leakage | □ Yes □ No |
| History of daytime incontinence after age 3.5y | □ Yes □ No |
| 2. Urinary frequency (≥ 8 times/d) | □ Yes □ No |
| 3. Urgency | □ Yes □ No |
| 4. Reduced voiding frequency (< 4 times/d) | □ Yes □ No |
| 5. Holding postures (e.g., leg crossing, heel pressing perineum) | □ Yes □ No |
| 6. Abdominal pressing to initiate urination | □ Yes □ No |
| 7. Interrupted stream or multiple voids | □ Yes □ No |
| 8. History of urinary tract infection | □ Yes □ No |
| 9. Urinary tract disease/malformation | □ Yes □ No |
| 10. Spinal/neurological disease | □ Yes □ No |
| Comorbidities (may affect treatment response) | |
| 1. Bowel symptoms or history (constipation resolution may improve enuresis) |  |
| Constipation (≤ 3 bowel movements/wk) | □ Yes □ No |
| Fecal incontinence | □ Yes □ No |
| 2. Psychological/behavioral/mental issues (e.g., ADHD, ASD) |  |
| Poor attention | □ Yes □ No |
| Hyperactivity | □ Yes □ No |
| Impulsivity | □ Yes □ No |
| Social interaction difficulties | □ Yes □ No |
| Narrow interests | □ Yes □ No |
| Repetitive behaviors | □ Yes □ No |
| 3. History of motor/learning/developmental disorders | □ Yes □ No |
| 4. Sleep assessment |  |
| Nighttime sleep duration | __________ |
| Wake-up time | __________ |
| Difficulty falling asleep, hard to wake, frequent dreams, daytime sleepiness | □ Yes □ No |
| Severe snoring or sleep apnea | □ Yes □ No |
| Drinking Habits |  |
| 1. Frequently drinks beverages | □ Yes  Amount and type:  □ No |
| 2. Drinks within 2 or 3 h before bedtime | □ Yes □ No |
| 3. Drinks more than 200 mL in the evening | □ Yes □ No |
| 4. Drinks milk or eats porridge/soup at dinner | □ Yes □ No |
| 5. Eats high-water fruits (e.g., watermelon) at night | □ Yes □ No |
| Family and Medical History |  |
| 1. Family history of enuresis (parents, siblings, other relatives) | □ Yes □ No |
| 2. History of urinary tract infection | □ Yes □ No |
| 3. History of spinal/urinary surgery | □ Yes □ No |
| 4. Use of medications affecting urination | □ Yes □ No |
| 5. Previous treatment for enuresis | □ No  □ Yes  Treatment method: |

*NMNE* non-monosymptomatic enuresis, *ADHD* attention deficit hyperactivity disorder, *ASD* autism spectrum disorder, *wk* week, *e.g.* for example, *etc* cetera.

Note This article has been previously published in *Journal of Clinical Pediatrics*, 2025, Volume 43, Issue 7, 2025, pages 483–499 (DOI: 10.12372/jcp.2025.25e0564); under the title “Chinese expert consensus on the management of nocturnal enuresis in children (2025)” in Chinese. *The Journal of Clinical Pediatrics* has graciously agreed to transfer the copyright of the English version of the “Chinese expert consensus on the management of nocturnal enuresis in children (2025)” to the *World Journal of Pediatrics*. The *Journal of Clinical Pediatrics* is an open access journal.

**Supplemental Table 2 Sample voiding diary (daytime)**

| Day 1 Date | | | | | Day 2 Date | | | | |
| --- | --- | --- | --- | --- | --- | --- | --- | --- | --- |
| Time | Fluid intake (mL) | Time | Urine volume (mL) | Incontinence (yes or no) | Time | Fluid intake (mL) | Time | Urine volume (mL) | Incontinence (yes or no) |
|  |  |  |  |  |  |  |  |  |  |
|  |  |  |  |  |  |  |  |  |  |
|  |  |  |  |  |  |  |  |  |  |
|  |  |  |  |  |  |  |  |  |  |
|  |  |  |  |  |  |  |  |  |  |
|  |  |  |  |  |  |  |  |  |  |
|  |  |  |  |  |  |  |  |  |  |
|  |  |  |  |  |  |  |  |  |  |
|  |  |  |  |  |  |  |  |  |  |
|  |  |  |  |  |  |  |  |  |  |

Note: reprinted or reproduced from *Journal of Clinical Pediatrics*, 2025, Volume 43, Issue 7, 2025, pages 483–499 (DOI: 10.12372/jcp.2025.25e0564); under the title “Chinese expert consensus on the management of nocturnal enuresis in children (2025)” by Chinese cooperative group for the management of pediatric enuresis, pediatric nephrology committee of Chinese Medical Doctor Association

**Supplemental Table 3 Sample voiding diary (Nighttime)**

| Characteristic | Day 1 | Day 2 | Day 3 | Day 4 | Day 5 | Day 6 | Day 7 |
| --- | --- | --- | --- | --- | --- | --- | --- |
| Sleep time |  |  |  |  |  |  |  |
| Wake time |  |  |  |  |  |  |  |
| Bedwetting |  |  |  |  |  |  |  |
| Bowel movement |  |  |  |  |  |  |  |
| Nighttime toilet visit |  |  |  |  |  |  |  |
| Nighttime urine volume (mL) |  |  |  |  |  |  |  |
| Diaper weight gain (g) |  |  |  |  |  |  |  |
| First morning urine volume (mL) |  |  |  |  |  |  |  |
| Total nighttime urine volume (mL) |  |  |  |  |  |  |  |

Note Reprinted or reproduced from *Journal of Clinical Pediatrics*, 2025, Volume 43, Issue 7, 2025, pages 483–499 (DOI: 10.12372/jcp.2025.25e0564); under the title “Chinese expert consensus on the management of nocturnal enuresis in children (2025)” by Chinese cooperative group for the management of pediatric enuresis, pediatric nephrology committee of Chinese Medical Doctor Association with permitted copyright.
